# Supplementary material for: Comparative Analysis of Embryonic Development and Mitochondrial Genome of a New Intergeneric Hybrid Grouper (Epinephelus fasciatus ♀ × Plectropomus leopardus ♂)
Source: Animals (Basel). 2025 Nov 28;15(23):3445. doi: 10.3390/ani15233445 (PMC12691364; doi:10.3390/ani15233445)
Supplement: Supplementary file 1 [file animals-15-03445-s001.zip › animals-3949236-supplementary.pdf]

Table S1. Mitochondrial genome characteristics of *Plectropomas leopardus*, *Epinephelus fasciatus*, and *E. fasciatus* ♀ × *P. leopardus* ♂.

Gene	Strand	*Plectropomus leopardus*			*Epinephelus fasciatus*			*E. Fasciatus* ♀ × *P. leopardus* ♂			Start_coden	Stop_coden
		Position (Start-End)	Length(bp)	Intergenic_spacer	Position (Start-End)	Length(bp)	Intergenic_spacer	Position (Start-End)	Length(bp)	Intergenic_spacer		
trnF(gaa)	F	1-69	69	-	1-70	70	-	1-70	70	-	-	-
rrnS	F	70-1018	949	0	71-1022	952	0	71-1022	952	0	-	-
trnV(tac)	F	1022-1092	71	3	1024-1094	71	1	1024-1094	71	1	-	-
rrnL	F	1096-2802	1707	3	1098-2798	1701	3	1098-2798	1701	3	-	-
trnL2(taa)	F	2800-2872	73	-3	2799-2873	75	0	2799-2873	75	0	-	-
nad1	F	2873-3847	975	0	2874-3848	975	0	2874-3848	975	0	ATG/ATG/ATG	TAA/TAA/TAA
trnI(gat)	F	3852-3921	70	4	3853-3922	70	4	3853-3922	70	4	-	-
trnQ(ttg)	R	3921-3991	71	-1	3922-3992	71	-1	3922-3992	71	-1	-	-
trnM(cat)	F	3992-4060	69	0	3993-4061	69	0	3993-4061	69	0	-	-
nad2	F	4061-5107	1047	0	4062-5108	1047	0	4062-5108	1047	0	ATG/ATG/ATG	TAA/TAA/TAA
trnW(tca)	F	5107-5177	71	-1	5108-5178	71	-1	5108-5178	71	-1	-	-
TrnA(tgc)	R	5179-5247	69	1	5180-5248	69	1	5180-5248	69	1	-	-
trnN(gtt)	R	5249-5321	73	1	5249-5321	73	0	5249-5321	73	0	-	-
trnC(gca)	R	5358-5424	67	36	5362-5428	67	40	5362-5428	67	40	-	-
trnY(gta)	R	5425-5494	70	0	5429-5499	71	0	5429-5499	71	0	-	-
cox1	F	5496-7046	1551	1	5501-7051	1551	1	5501-7051	1551	1	GTG/GTG/GTG	TAA/TAA/TAA
trnS2(tga)	R	7047-7117	71	0	7053-7123	71	1	7053-7123	71	1	-	-
trnD(gtc)	F	7120-7193	74	2	7124-7197	74	0	7124-7197	74	0	-	-
cox2	F	7200->7890	691	6	7205->7895	691	7	7205->7895	691	7	ATG/ATG/ATG	T/T/T
trnK(ttt)	F	7891-7965	75	0	7896-7968	73	0	7896-7968	73	0	-	-

atp8	F	7967-8134	168	1	7970-8137	168	1	7970-8137	168	1	ATG/ATG/ATG	TAA/TAA/TAA
atp6	F	8188-8808	621	53	8155-8811	657	17	8155-8811	657	17	ATG/ATA/ATA	TAA/TAA/TAA
cox3	F	8808-9593	786	-1	8811-9596	786	-1	8811-9596	786	-1	ATG/ATG/ATG	TAA/TAA/TAA
trnG(tcc)	F	9593-9662	70	-1	9596-9666	71	-1	9596-9666	71	-1	-	-
nad3	F	9663-10013	351	0	9667->10015	349	0	9667->10015	349	0	ATG/ATG/ATG	TAG/T/T
trnR(tcg)	F	10012-10080	69	-2	10016-10084	69	0	10016-10084	69	0	-	-
nad4l	F	10081-10377	297	0	10085-10381	297	0	10085-10381	297	0	ATG/ATG/ATG	TAA/TAA/TAA
nad4	F	10371->1175 1	1381	-7	10375->11755	1381	-7	10375->11755	1381	-7	ATG/ATG/ATG	T/T/T
trnH(gtg)	F	11752-11820	69	0	11756-11825	70	0	11756-11825	70	0	-	-
trnS1(gct)	F	11821-11898	78	0	11826-11895	70	0	11826-11895	70	0	-	-
trnL1(tag)	F	11905-11977	73	6	11911-11983	73	15	11911-11983	73	15	-	-
nad5	F	11978-13816	1839	0	11984-13822	1839	0	11984-13822	1839	0	ATG/ATG/ATG	TAA/TAA/TAA
nad6	R	13813-14334	522	-4	13819-14340	522	-4	13819-14340	522	-4	ATG/ATG/ATG	TAG/TAA/TAA
trnE(ttc)	R	14335-14403	69	0	14341-14409	69	0	14341-14409	69	0	-	-
cob	F	14406->1554 6	1141	2	14417->15557	1141	7	14417->15557	1141	7	ATG/ATG/ATG	T/T/T
trnT(tgt)	F	15547-15618	72	0	15558-15630	73	0	15558-15630	73	0	-	-
trnP(tgg)	R	15618-15688	71	-1	15630-15699	70	-1	15630-15699	70	-1	-	-
